# The Enhanced Adhesion of Eosinophils Is Associated with Their Prolonged Viability and Pro-Proliferative Effect in Asthma

**DOI:** 10.3390/jcm8091274

**Published:** 2019-08-22

**Authors:** Andrius Januskevicius, Ieva Janulaityte, Virginija Kalinauskaite-Zukauske, Reinoud Gosens, Kestutis Malakauskas

**Affiliations:** 1 Laboratory of Pulmonology, Department of Pulmonology, Lithuanian University of Health Sciences, LT-44307 Kaunas, Lithuania; 2 Department of Pulmonology, Lithuanian University of Health Sciences, LT-44307 Kaunas, Lithuania; 3 Department of Molecular Pharmacology, University of Groningen, 9713 AV Groningen, The Netherlands

**Keywords:** eosinophil, adhesion, viability, proliferation, airway smooth muscle cell, pulmonary fibroblast, phenotype, asthma

## Abstract

Before eosinophils migrate into the bronchial lumen, they promote airway structural changes after contact with pulmonary cells and extracellular matrix components. We aimed to investigate the impact of eosinophil adhesion to their viability and pro-proliferative effect on airway smooth muscle (ASM) cells and pulmonary fibroblasts during different asthma phenotypes. A total of 39 individuals were included: 14 steroid-free non-severe allergic asthma (AA) patients, 10 severe non-allergic eosinophilic asthma (SNEA) patients, and 15 healthy control subjects (HS). For AA patients and HS groups, a bronchial allergen challenge with *Dermatophagoides pteronysinnus* was performed. Individual combined cells cultures were prepared between isolated peripheral blood eosinophils and ASM cells or pulmonary fibroblasts. Eosinophil adhesion was measured by evaluating their peroxidase activity, cell viability was performed by annexin V and propidium iodide staining, and proliferation by Alamar blue assay. We found that increased adhesion of eosinophils was associated with prolonged viability (*p* < 0.05) and an enhanced pro-proliferative effect on ASM cells and pulmonary fibroblasts in asthma (*p* < 0.05). However, eosinophils from SNEA patients demonstrated higher viability and inhibition of pulmonary structural cell apoptosis, compared to the AA group (*p* < 0.05), while their adhesive and pro-proliferative properties were similar. Finally, in the AA group, in vivo allergen-activated eosinophils demonstrated a higher adhesion, viability, and pro-proliferative effect on pulmonary structural cells compared to non-activated eosinophils (*p* < 0.05).

## 1. Introduction

Chronic eosinophilic inflammation is a major factor in the development of airway remodeling in asthma [1]. The contribution of various cytokine networks makes asthma pathophysiology very complex, and these mechanisms vary between patients with different asthma phenotypes. Eosinophils are a significant source of cytokines, chemokines, growth factors, and enzymes [2], therefore, their increased infiltration into asthmatic lungs leads to the disturbance of normal lung homeostasis [3].

Eosinophils develop from bone marrow progenitors under the control of a dedicated set of transcription factors and the cytokines interleukin (IL)-3, IL-5, and granulocyte-macrophage colony-stimulating factor (GM-CSF) [4]. Eosinophils’ half-life is between 3 to 24 h in peripheral blood [5], while being in the lungs prolongs their half-life up to 36 h [6]. Survivability-promoting signals can be different. IL-5 is the most important and specific survival factor for eosinophils [7], but important mediators also include GM-CSF and IL-3 [8], tumor necrosis factor-α [9], leptin [10], and cluster of differentiation 40 engagement [11]. It is known that combined culturing with pulmonary structural cells promotes eosinophils survival [12,13,14,15], however, the precise mechanisms remain unknown. Integrins as transmembrane molecular mechanosensors may change their activation states under asthmatic conditions and transduce the signal through the cytoskeleton, thus regulating eosinophil activity and viability [16,17].

In addition to chronic inflammation, both allergic and non-allergic phenotypes of asthma are characterized by structural changes in the lungs, which is called airway remodeling [18]. Airway remodeling in asthma includes epithelial changes, subepithelial and airway smooth muscle (ASM) thickening, as well as bronchial neoangiogenesis that develops after repetitive cycles of tissue injury and abnormal repair processes because of chronic inflammation [19]. Airway remodeling develops mainly because of disturbed ASM cells and pulmonary fibroblast proliferation that determine the increase in tissue mass due to enhanced cell numbers and the release of the extracellular matrix (ECM) [20]. Studies demonstrate that direct interaction with eosinophils promotes pulmonary fibroblasts and ASM cell proliferation [21,22,23,24]. Eosinophil-released mediators, such as transforming growth factor β-1 and cysteinyl leukotrienes, are the main ASM cells, as well as being pulmonary fibroblast proliferation- and differentiation-promoting factors [25,26]; however, more research is needed to understand increased eosinophil activity or prolonged viability dominates in their pro-proliferative effect.

Asthma severity correlates with peripheral blood and sputum eosinophils count [27]. However, as major symptoms and airway structural changes between allergic and non-allergic asthma phenotypes overlap, the pathways through which the disease develops and how eosinophils are involved in the disease pathogenesis are different [28]. This highlights the hypothesis that further course of the disease during different asthma phenotypes or during disease exacerbation can be predicted not only by increased eosinophil count but also by their different biological properties.

We hypothesized that adhesion of eosinophils may determine their survivability properties and further effect on pulmonary structural changes in asthma. Isolated blood eosinophils demonstrate the biological role closely related to lung eosinophil functions because they are mainly activated in the bone marrow under the maturation and in peripheral blood by inflammatory mediators [29,30]. Therefore, our use of a combined cell culture model between blood eosinophils and pulmonary structural cells allows for imitation of in vivo processes. We evaluated the effect of adhesion to pulmonary structural cells on eosinophil viability and pro-proliferative effects for different asthma phenotypes. Moreover, we investigated, in vivo, how a provoked acute allergic asthma episode after a bronchial allergen challenge affects eosinophil activity.

## 2. Experimental Section

### 2.1. Ethics Statement

The study protocol was approved by the Regional Biomedical Research Ethics Committee of the Lithuanian University of Health Sciences (BE-2-13). Each participant was informed about the ongoing investigation and gave his/her written consent. Trial registration: ClinicalTrials.gov Identifier NCT03388359.

### 2.2. Study Population

The study population consists of only newly recruited, not studied individuals.

The study included steroid-free allergic asthma (AA) patients, severe non-allergic eosinophilic asthma (SNEA) patients with high doses of inhaled steroids, and healthy non-smoking subjects (HS) who comprised the control group. The participants were men and women between the ages of 18–50 years old who signed written informed consent. The patients were recruited from the Department of Pulmonology at the Hospital of the Lithuanian University of Health Sciences Kaunas Clinics.

The AA group were newly-established and untreated non-severe patients, approved with symptoms and medical history for at least a 12 month period, having a positive skin prick test to *Dermatophagoides pteronyssinus* allergen and positive bronchial challenge with methacholine.

The SNEA group was a non-allergic phenotype, approved by negative skin prick tests, and with asthma diagnosis for at least 1 year. Peripheral eosinophil counts were ≥0.3 × 10^9^/L during the screening visit or ≥0.15 × 10^9^/L if there was a documented eosinophil count ≥0.3 × 10^9^/L in the 12 month period before the screening. A severe course of the disease was approved with at least 12 month treatment of high doses of inhaled steroids combined with long-acting beta-agonist ± long-acting antimuscarinic agent ± episodic use of oral corticosteroids.

The HS group was without allergic and other chronic respiratory diseases.

Exclusion criteria for all groups were clinically significant permanent allergy symptoms, active airway infection 1 month prior the study, exacerbation ≤ 1 month prior to study, use of oral steroids ≤ 1 month prior to study, and smoking.

### 2.3. Study Design

Initially, all study subjects underwent physical examination, spirometry, a methacholine challenge test, and a skin prick test to verify the inclusion and exclusion criteria. If individuals matched the criteria, they were informed about the requirements for participation and informed written consent was obtained.

At the first visit of the study, peripheral blood was collected and measured for exhaled fractional exhaled nitric oxide (FeNO). Isolated peripheral blood eosinophils were counted, assessed in their viability, and immediately prepared in combined cell cultures with ASM cells and pulmonary fibroblasts. Additionally, AA patients and HS after primary collection of peripheral blood underwent a bronchial allergen challenge with *D. pteronyssinus* allergen.

**Figure 1:**
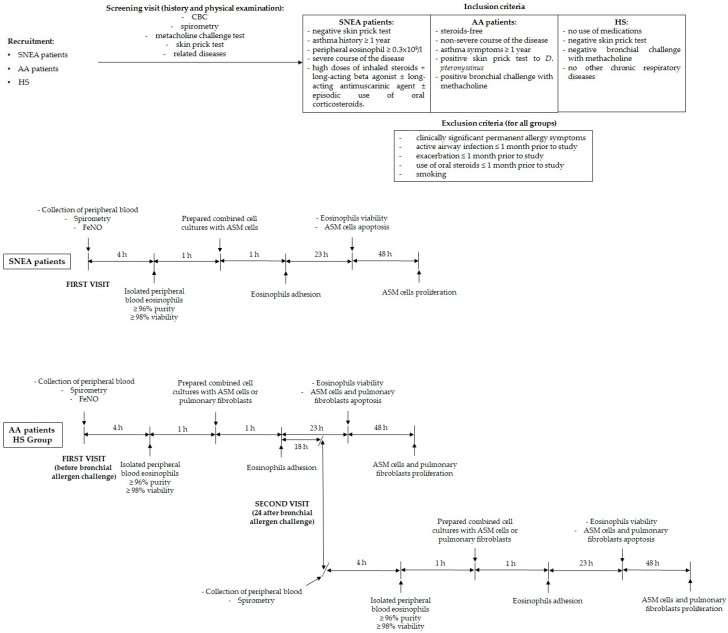
Experimental study design. CBC—Complete blood count; SNEA—Severe non-allergic eosinophilic asthma; AA—Allergic asthma; HS—Healthy subjects; FeNO—Fractional exhaled nitric oxide; ASM—Airway smooth muscle.

The second visit was 24 h after a bronchial allergen challenge for subjects whom this test was performed, and all procedures were repeated according to the first visit. The experimental study design is represented in Figure 1.

Eosinophil count (<1.5 × 10^6^/20 mL blood), viability (<98%), and purity (<96%) after their isolation processes, as well as eosinophil adhesion intensity (equal to control value) was used as experimental exclusion criteria for all investigated subjects. All data provided in the manuscript were from subjects who passed these criteria.

We performed the unblinded type of experiments, as well-planned preparation before the recruitment of each study subjects was required, and the whole experimental plan was performed in the same week.

### 2.4. Lung Function Testing

Pulmonary function was tested by using an ultrasonic spirometer (Ganshorn Medizin Electronic, Niederlauer, Germany). Baseline forced expiratory volume in 1 s (FEV_1_), forced vital capacity (FVC), and FEV_1_/FVC ratio were recorded as the highest of three reproducible measurements. The results were compared with the predicted values matched for age, body height, and sex according to the standard methodology.

Airway responsiveness was assessed using inhaled methacholine via pressure dosimeter (ProvoX, Ganshorn Medizin Electronic, Niederlauer, Germany). Aerosolized methacholine was inhaled at 2 min intervals, starting with a 0.0101 mg methacholine dose, and increasing it by steps up to 0.121, 0.511, and 1.31 mg of the total cumulative dose was achieved, or until a 20% decrease in FEV_1_ from the baseline. The bronchoconstriction effect of each dose of methacholine was expressed as a percentage of decrease in FEV_1_ from the baseline value. The provocative dose of methacholine causing a ≥20% fall in FEV_1_ (PD_20M_) was calculated from the log dose-response curve by linear interpolation of two adjacent data points.

### 2.5. Skin Prick Testing

All patients were screened for allergy by the skin prick test using standardized allergen extracts (Stallergenes, S.A., France) for the following allergens: *D. pteronyssinus*, *Dermatophagoides farinae*, cat and dog dandruff, five mixed grass pollens, birch pollen, mugwort, *Alternaria*, *Aspergillus*, and *Cladosporium*. Diluent (saline) was used for the negative control, and histamine hydrochloride (10 mg/mL) was used for the positive control. Skin testing was read 15 min after application. The results of the skin prick test were considered positive if the mean wheal diameter was higher than 3 mm.

### 2.6. FeNO Measurement

All study subjects underwent fractional exhaled nitric oxide (FeNO) analysis with an on-line method using a single breath exhalation and an electrochemical assay (NIOX VERO, Circassia, UK), according to European Respiratory Society—American Thoracic Society guidelines. Patients made an inspiration of FeNO-free air via a mouthpiece, immediately followed by full exhalation at a constant rate (50 mL/s) for at least 10 s. The mean of three readings at the end of the expiration (plateau phase) was taken as the representative value for each measurement. Values that were 12 ppb or more were considered elevated values, according to ATS-ERS criteria.

### 2.7. Bronchial Allergen Challenge Test

Inhaled *D. Pteronyssinus* allergen (DIATER, Spain) was delivered via pressure dosimeter (ProvoX, Ganshorn Medizin Electronic, Niederlauer, Germany). The starting point for the assessment of bronchoconstriction effect was 2 min after nebulized saline inhalation. The aerosolized allergen was inhaled at 10 min intervals, starting with 0.1 histamine equivalent prick (HEP)/mL allergen concentration, increasing it by steps up to 1.0, 10.0, 20.0, 40.0, 60.0 HEP/mL, or if a 20% decrease in FEV_1_ from the baseline was achieved. The provocative dose of allergen causing a ≥20% fall in FEV_1_ (PD_20A_) was calculated from the log dose-response curve by linear interpolation of two adjacent data points.

### 2.8. Analysis of Peripheral Blood Cells

The peripheral blood analysis for the complete blood count test was performed on an automated hematology analyzer XE-5000™ (Sysmex, Kobe, Japan).

### 2.9. Granulocyte Isolation from Human Peripheral Blood and Eosinophil Enrichment

**Figure 2:**
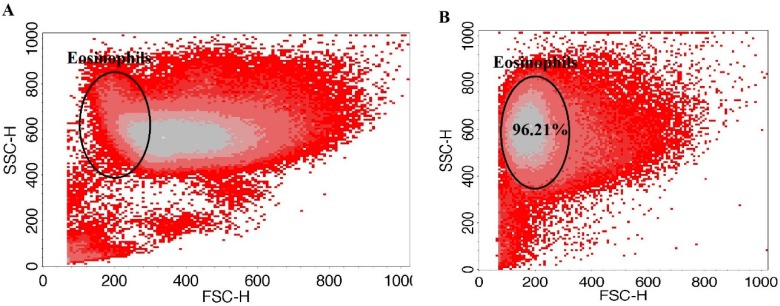
Eosinophil enrichment. (**A**) peripheral blood granulocytes after high-density centrifugation and erythrocyte lysis. (**B**) peripheral blood eosinophils after negative magnetic labeling and separation. Collected cell number—1 × 10^5^. Eosinophil quantity expressed from total collected cells counts rejecting debris.

Approximately 24 mL of peripheral blood was collected into sterile Ethylenediaminetetraacetic acid-containing vacutainers (BD Bioscience, San Jose, CA, USA). The whole blood was then diluted with 1x phosphate-buffered saline (PBS) (GIBCO, Paisley, UK) up to 50 mL and mixed thoroughly. The whole blood was layered on Ficoll-Paque PLUS (GE Healthcare, Helsinki, Finland) and centrifuged at 400× *g* force for 30 min at room temperature. The supernatant was removed and the bottom-most layer comprising the granulocyte fraction of cells and erythrocytes was collected. Then, hypotonic lysis of erythrocytes was performed. Into the tubes with cells, half volume of sterile water was added and gently mixed for no longer than 10 s, immediately supplementing the mixture with an equal volume of 2x concentrated PBS and centrifuged at 300× *g* force for 10 min. The procedure was repeated until no red blood cells were left. Then, cells were counted and viability evaluated using an ADAM automatic cell counter (Witec AG, Switzerland). Eosinophil enrichment was performed by negative selection from the granulocyte fraction using Magnetic-activated cell sorting (MACS)magnetically-labeled MicroBeads (Miltenyi Biotec, Somerville, MA, USA). The manufacturer confirms that eosinophil separation kits do not influence eosinophil viability, and that separation efficiency is more than 96%. MACS buffer (containing 0.5% bovine serum albumin (BSA) and 2 mM EDTA in PBS, with a pH of 7.2) was prepared by diluting MACS BSA stock solution 1:20 in autoMACS rinsing solution. The granulocyte pellet was resuspended in cold MACS buffer (40 μL per 1 × 10^7^ cells) and incubated with biotin antibody cocktail (biotin-conjugated monoclonal antibodies against CD2, CD14, CD16, CD19, CD56, CD123, and CD235a (glycophorin A)) (10 μL per 1 × 10^7^ cells) for 10 min. After incubation, 20 μL of anti-biotin microbeads (microbeads conjugated to monoclonal mouse anti-biotin immunoglobulin(Ig)-G1 per 1 × 10^7^ cells were added, mixed, and incubated for an additional 15 min at 4 °C. An large separation column (Miltenyi Biotec, USA) was prepared during this time by placing the column in the magnetic field of a MACS separator and washing it with 2 mL of MACS buffer. A pre-separation filter (30 μm; Miltenyi Biotec) was rinsed with MACS buffer and placed on top of the column. The cells were then applied to the pre-separation filter/LS column, and the magnetically labeled non-eosinophils were retained on the column in the magnetic field of the separator while the unlabeled eosinophils passed through the column. Cells were eluted with 5 mL of MACS buffer and centrifuged (300× *g*, 10 min, 4 °C), and the pellet was resuspended in 5 mL of PBS. Eosinophils were counted using an ADAM automatic cell counter. To check eosinophil purity after magnetic separation, a diluted eosinophil suspension was analyzed by flow cytometer FacsCalibur (BD, USA) according to forward and side light scattering to determine whether there were any other cells in the suspension (Figure 2). As an internal control, after usage of the new isolation kit, eosinophil purity was tested with alternative May-Grunwald Giemsa staining and inspected by light microscopy.

### 2.10. Combined Cell Cultures between Isolated Eosinophils and ASM Cells or Pulmonary Fibroblasts

Individual combined cell cultures (co-cultures) of eosinophils and pulmonary structural cells were prepared for all experiments. We used healthy human ASM cells, immortalized by stable expression of human telomerase reverse transcriptase as described in [31] and a commercial MRC-5 (Sigma, Ronkonkoma, NY, USA) lung fibroblast line. The main cell line was cultivated under standard culture conditions of 5% CO_2_ in air at 37 °C with medium renewal every 3 days. For all experiments, passaged ASM cells were grown on plastic dishes in Dulbecco’s modified Eagle’s medium (DMEM) (GIBCO by Life Technologies, UK) and MRC-5 pulmonary fibroblasts in minimum essential Eagle’s medium (EMEM) (GIBCO, Paisley, UK), supplemented with streptomycin/penicillin (2% *v*/*v*; Pen-Strep, GIBCO by Life Technologies, Paisley, UK), amphotericin B (1% *v*/*v*; GIBCO, Paisley, UK), and fetal bovine serum (10% *v*/*v*; GIBCO by Life Technologies). After reaching sufficient confluence, cells were passaged by trypsinization. Cells were serum-deprived in respective medium, supplemented with antibiotics and insulin, transferrin, and selenium reagent (GIBCO by Life Technologies) before each experiment to stop cell proliferation and avoid possible errors due to the effect of mediators in serum. The same lines of ASM and MRC-5 cells were used for whole investigating subjects. Avoiding a decrease in cell activity and viability after repeated times of passage, the new cells of the mainline were unfrozen every time after six passages.

**Figure 3:**
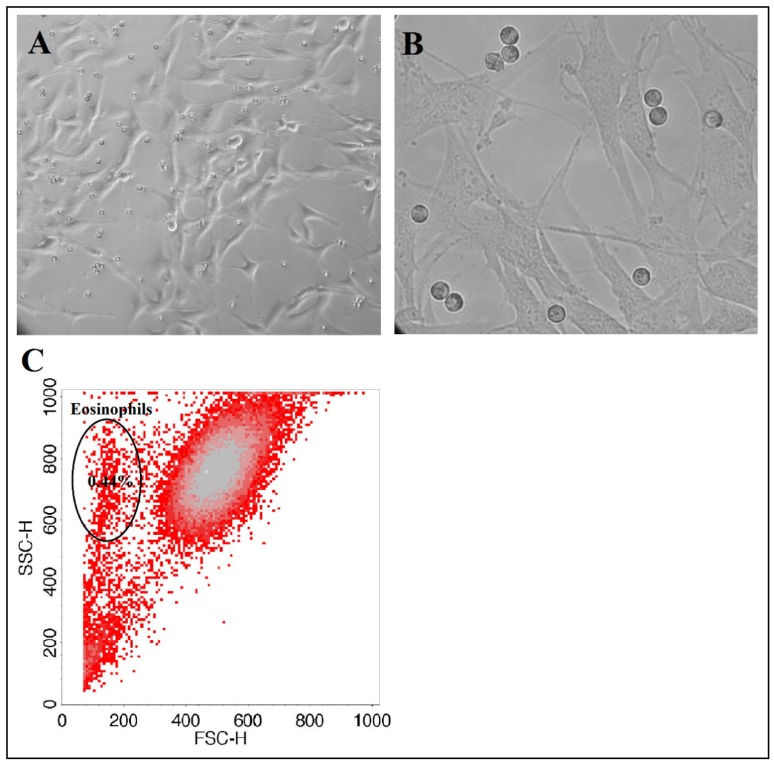
Combined cell cultures between eosinophils and ASM cells. (**A**) the picture at 10× objective. (**B**) the picture at 40× objective. (**C**) remaining eosinophil number after combined cell culture separation. Collected cell number—1 × 10^5^. Eosinophil number is expressed from total collected cell count, rejecting cell debris.

ASM and MRC-5 cells were grown in six-well (confluency approximately 16 × 10^4^ cells) or 24-well (confluency approximately 4 × 10^4^ cells) culture plates (CytoOne, StarLab, Brussels, Belgium.). Co-cultures with isolated eosinophils were prepared by adding 5 × 10^4^ or 1.25 × 10^4^ of eosinophils, respectively. For observing and visualization of cell growth and co-cultures, we used an inverted microscope (CETI Inverso TC100, Medline Scientific, Chalgrove, UK) with a 10×/22 mm wide-field eyepiece and phase contrast 10×/0.25 objective and an installed XM10-IR-2 camera (Olympus, Tokyo, Japan) (Figure 3).

At the study day, experiments were divided into different groups, according to the growth medium supplements. Investigated individual blood serum—2% of *v*/*v*—was used to maintain further eosinophil activation after isolation processes and to verify if the eosinophils were isolated in their most activated form, whilst 25 nm of vitamin C (ascorbic acid) was used as the most accessible natural antioxidant to eliminate eosinophil-released reactive oxygen species and verify its effect on the proliferation of pulmonary structural cells.

### 2.11. Eosinophils Adhesion Assay

ASM or MRC-5 cells were seeded in 24-well plates and grown for 3 days in 5% CO_2_ at 37 °C until a confluency of approximately 4 × 10^4^ cells. Then, the medium was removed, and wells were washed twice with warm PBS. The medium was changed 24 h before the experiments by adding serum-free medium, supplemented with 1% insulin-transferrin-selenium reagent. Eosinophil adhesion was measured after 1 h of incubation, which is a sufficient period for eosinophils to adhere in co-culture [22]. After incubation, non-adhered eosinophils were removed, and the remaining cells were washed twice with warm PBS. Eosinophil adhesion was determined by measuring residual eosinophil peroxidase (EPO) activity as described [32]. Because intercellular EPO levels were identified as being decreased in eosinophils from asthmatic individuals owing to degranulation [33], we normalized it by preparing a calibration curve of fixed eosinophil count EPO activity for each experiment. To assay EPO activity, 116 μL of DMEM medium without phenol red and 116 μL of EPO substrate (1 mM H_2_O_2_, 1 mM *o*-phenylenediamine, and 0.1% Triton X-100 in Tris buffer, pH 8.0) were added to each well. After 30 min at 37 °C, 68 μL of 4 M H_2_SO_4_ was added to each well to stop the reaction and the absorbance was measured at 490 nm by a microplate reader. Results were expressed as % of adhered eosinophil number from max added, calculated from a calibration curve. Added eosinophil number—1.25 × 10^4^ (double amount—2.5 × 10^4^).

### 2.12. Cell Viability Assay

Viability of ASM cells, pulmonary fibroblasts, and eosinophils were performed by fluorescent staining with annexin V for apoptotic cells and propidium iodide (PI) for necrotic cells.

ASM cells and pulmonary fibroblasts were grown in six-well plates until confluency of approximately 16 × 10^4^ cells. On the day of experiments, a co-culture with 5 × 10^4^ of isolated eosinophils was prepared in the serum-free growth medium, or medium supplemented with 2% (*v*/*v*) of investigated subjects’ blood serum. After 24 h of co-culturing, used eosinophils were collected into 15mL centrifuge tubes (Corning Inc., New York, NY, USA), together with eosinophils incubated alone at the same conditions. Then, ASM cells and pulmonary fibroblasts were trypsinized, collected and, together with eosinophils, centrifuged at 400× *g* for 10 min.

For the cell viability assay, we used an fluorescein isothiocyanate (FITC) Annexin V Apoptosis Detection Kit II (BD Bioscience, San Jose, CA, USA) and adapted the method according to the manufacturer’s recommendation. Before every experiment, we used additional controls of unstained cells, cells stained with FITC annexin V (no PI), and cells stained with PI (no FITC annexin V). For the viability assay of structural cells as a control, we used ASM cells or pulmonary fibroblasts without co-culturing with eosinophils. Eosinophil’s effect was compared with normal ASM and pulmonary fibroblast apoptosis in the serum-free growth medium, but was supplemented with an insulin-transferrin-selenium compound to maintain normal conditions.

For the viability assay of eosinophils, we normalized the data according to the results received from the centrifuged growth medium, which was used in the control structural cell cultures without co-culturing with eosinophils and excluding possible errors from cellular debris. Eosinophils and structural cells significantly differ in size and granularity, therefore, appropriate gating on forward and side scattering excludes any remaining culture heterogeneity.

### 2.13. Alamar Blue Proliferation Assay

Cells for proliferation measurements were grown in 24-well plates in conditions described previously in fetal bovine serum-supplemented growth medium until confluency of approximately 4 × 10^4^ cells/well. Growth medium was changed into the serum-free medium 24 h before the experiments. ASM cells or pulmonary fibroblasts were co-cultured with a respective group of eosinophils isolated from AA patients, SNEA patients, or the HS for 72 h. Thereafter, all cells were washed twice with warm PBS, plates were gently smashed in the middle of the plate to detach residual eosinophils who were significantly more weakly adhered compared with structural cells, and again were washed twice with warm PBS. Proliferation was evaluated by incubating wells with Hank’s balanced salt solution containing Alamar blue solution (10% *v*/*v*; Invitrogen by Life Technologies, Paisley, UK). Conversion of Alamar blue to the reduced form was dependent on the metabolic activity of structural cells and was assayed by dual-wavelength spectrophotometry at wavelengths of 570 nm and 600 nm. As indicated by the manufacturer, the degree of Alamar blue conversion is proportional to the number of viable cells. The data are expressed as the percent increase or decrease in Alamar blue conversion by ASM cells or pulmonary fibroblasts compared with control cells (without co-culturing with eosinophils), which did not proliferate during the culturing period because serum-free growth medium were used. Added eosinophils number—1.25 × 10^4^; 2x amount—2.5 × 10^4^; 1/2x amount—0.6 × 10^4^. Used investigated subjects blood serum volume—2% *v*/*v*; ascorbic acid concentration—25 nM.

### 2.14. Statistical Analysis

Statistical analysis was performed with GraphPad Prism 6 for Windows (ver. 6.05, 2014; GraphPad Software Inc., San Diego, CA, USA). Significant differences between two independent groups were determined using the Mann–Whitney two-sided U-test. The Wilcoxon matched-pairs signed-rank two-sided test was used for dependent groups. Minimum limit for statistically significant values—*p* < 0.05.

## 3. Results

### 3.1. Characteristics of the Studied Participants

We investigated 39 nonsmoking adults (14 men and 25 women): 14 steroid-free non-severe allergic asthma (AA) patients, 10 severe non-allergic eosinophilic asthma (SNEA) patients, and 15 healthy non-smoking control subjects (HS). The main characteristics of the study participants are shown in Table 1. The highest eosinophils count was observed in the SNEA group, however, in AA patients, it was also increased compared with HS. The IgE levels were significantly increased in AA and SNEA patients, compared with HS, but the highest level was in the AA group. FeNO was equally increased in both the AA and SNEA groups, compared with HS. Moreover, at the baseline, significant deterioration of lung function was observed only in SNEA patients.

### 3.2. Eosinophil Adhesion and Survivability

**Figure 4:**
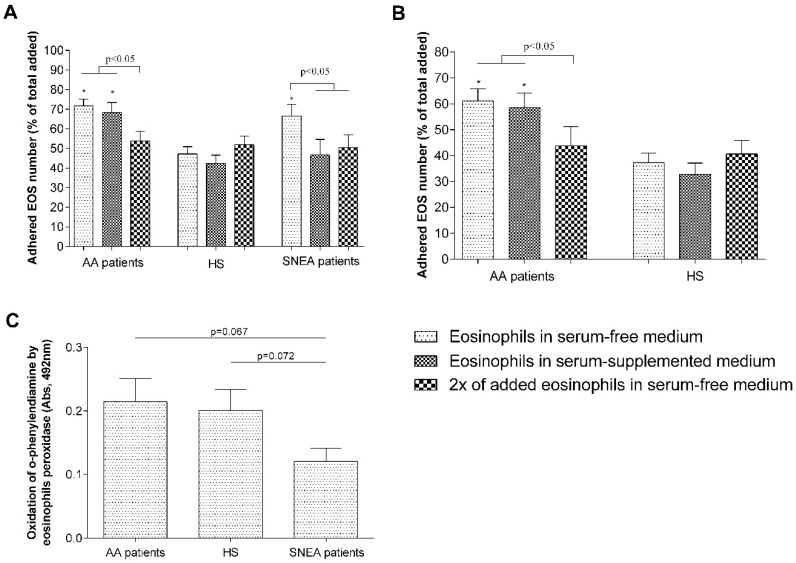
The efficiency of eosinophil adhesion. (**A**) Eosinophil adhesion in co-culture with airway smooth muscle (ASM) cells; (**B**) eosinophil adhesion in co-culture with pulmonary fibroblasts. (**C**) Eosinophil peroxidase (EPO) substrate activity of 12,500 eosinophils. EOS—eosinophils, AA—Allergic asthma, SNEA—Severe non-allergic eosinophilic asthma, HS—Healthy subjects. Results from independent experiments of AA—*n* = 14; HS—*n* = 15; SNEA—*n* = 10. * *p* < 0.05 compared with adhesion of the healthy eosinophil group. Added blood serum: 2% of V/V. Statistical analysis: between investigated groups—Mann–Whitney two-sided *U*-test; within one study group—Wilcoxon matched-pairs signed-rank two-sided test.

We observed that 71.7% ± 3.5% of AA patients’ and 66.6% ± 5.8% of SNEA patients’ eosinophils adhere onto the surface of ASM cells after 1 h of incubation, and this count was significantly increased compared to healthy eosinophils (47.2% ± 3.7%, *p* < 0.05) (Figure 4A). Supplementing growth medium with investigated individuals’ blood serum had a significant negative effect only in the SNEA group—the adhered eosinophils ratio decreased to 46.7% ± 7.9% of the added eosinophils count, *p* < 0.05. Moreover, by using double the amount of added eosinophils in the culture well, we received a significant (*p* < 0.05) decrease in the adhered eosinophils ratio in the AA and SNEA groups to 53.8% ± 5.0% and 50.5% ± 6.4% of the total added eosinophil count, respectively, with no differences in the HS group (Figure 4A). Similar results were obtained by measuring AA and HS eosinophil adhesion to pulmonary fibroblasts—adhered eosinophils’ number in the AA group was 61.2% ± 4.6%, and 37.3% ± 3.7% in the HS group, *p* < 0.05. Significant differences after supplementing the medium with blood serum were not observed in both groups. Moreover, in the AA group, the adhered eosinophil ratio decreased to 43.8% ± 7.4% after using double the amount of added eosinophils (*p* < 0.05) (Figure 4B).

Moreover, we observed that AA and HS eosinophils’ peroxidase activity, evaluated as the ability to oxidize o-phenylenediamine in the presence of their peroxidases, are similar—The average absolute value values of oxidized o-PD was 0.21 ± 0.04 optical density (O.D) and 0.2 ± 0.3 O.D, respectively. The average SNEA eosinophil peroxidase activity was only 0.12 ± 0.02 O.D; however, there was no statistical significance upon comparison with AA and HS groups, *p* = 0.069 and 0.073, respectively (Figure 4C).

**Figure 5:**
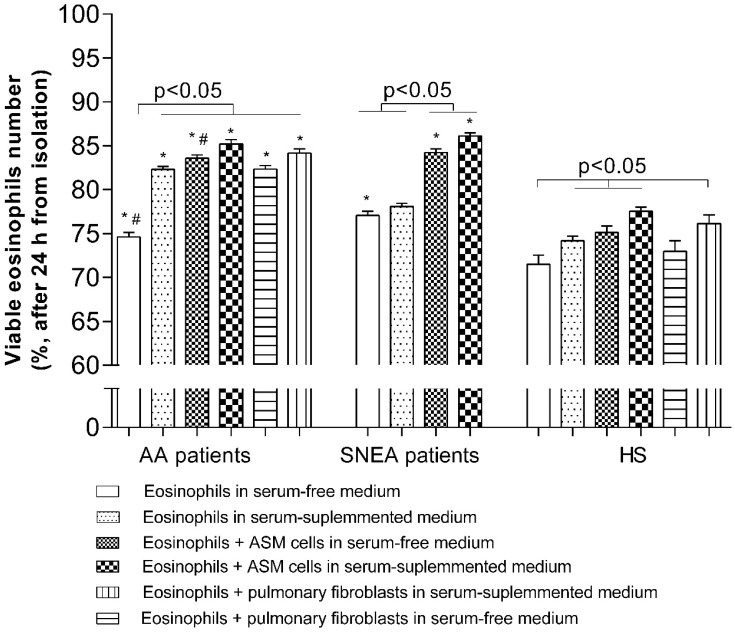
Eosinophil viability. Changes in the number of viable eosinophils during different incubation conditions. Results from independent experiments of AA—*n* = 14, HS—*n* = 15, SNEA—*n* = 10; AA—Allergic asthma, SNEA—Severe non-allergic eosinophilic asthma, HS—Healthy subjects. * *p* < 0.05 compared with the viability of the healthy eosinophil group; ^#^ *p* < 0.05 compared with the viability of the SNEA eosinophil group; Added blood serum—2% of *v*/*v*. Statistical analysis: between investigated groups—Mann–Whitney two-sided U-test; within one study group—Wilcoxon matched-pairs signed-rank two-sided test.

Furthermore, we investigated the eosinophil adhesion effect on their survivability. We observed that 71.5% ± 1.1% of HS eosinophils are still viable after 24 h of incubation in serum-free growth medium, and that this amount is significantly lower when compared with the viability of AA and SNEA patients’ eosinophils at 74.6% ± 0.5% and 77.1 ± 0.5%, respectively. The number of viable eosinophils increased significantly to 82.3% ± 0.4% in the AA group and to 74.2% ± 0.5% in the HS group when the medium was supplemented with 2% of *v*/*v* of the investigated subjects‘ blood serum (*p* < 0.01), but it had no effect on the SNEA patient group. Making co-cultures with ASM cells in serum-free medium had the same effect as blood serum—In the AA group, the number of viable eosinophils increased to 83.6% ± 0.4%; in the SNEA group, to 84.2% ± 0.4%; in the HS group, to 75.1% ± 0.8% (*p* < 0.05), and this effect in AA and SNEA groups was significantly greater compared with HS (*p* < 0.05). A similar effect was obtained when co-cultures of eosinophils were taken from AA and HS groups with pulmonary fibroblasts–viability increased to 82.3% ± 0.4% and 73.0% ± 1.2%, respectively, (*p* < 0.05). Supplementing co-culture growth medium with investigated subjects’ blood serum did not have any additional effect on the viability of eosinophils (Figure 5).

### 3.3. Eosinophil Effect on Pulmonary Structural Cell Proliferation and Apoptosis

**Figure 6:**
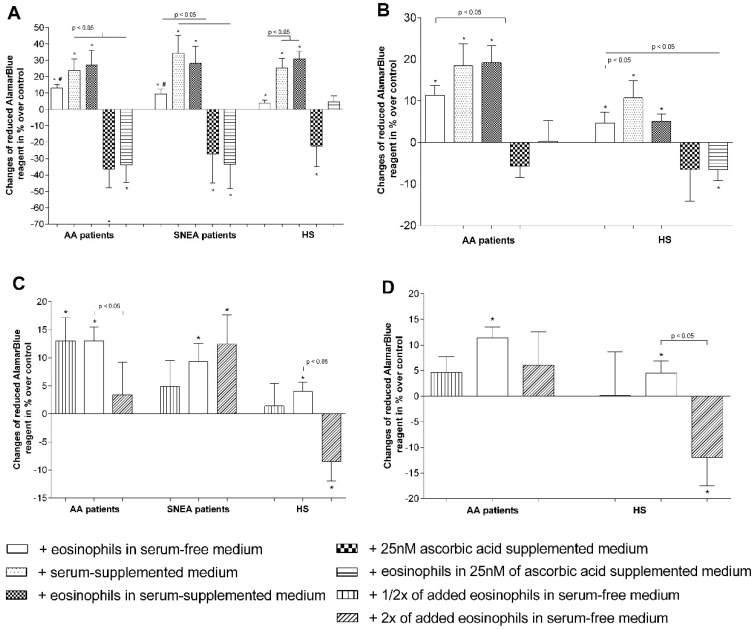
Eosinophils’ effect on pulmonary structural cell proliferation. (**A**) Eosinophils’ effect on ASM cell proliferation; (**B**) eosinophils’ effect on pulmonary fibroblast proliferation; (**C**) the effect of adding a different eosinophil count on ASM cell proliferation; (**D**) the effect of adding a different eosinophil count on pulmonary fibroblast proliferation. Results from independent experiments of AA—*n* = 14, HS—*n* = 15, SNEA—*n* = 10. * *p* < 0.05 compared with control ASM cells or pulmonary fibroblasts without co-culturing with eosinophils, ^#^ *p* < 0.05 compared with the healthy subject group. Added eosinophils count—1/2x = 6,250, 1x = 12,500, 2x = 25,000. Added blood serum—2% of *v*/*v*. Ascorbic acid concentration—25 nM. Statistical analysis: between investigated groups—Mann–Whitney two-sided U-test; within one study group—Wilcoxon matched-pairs signed-rank two-sided test.

After 72 h of co-culturing, eosinophils promoted ASM cell proliferation by 13.0% ± 2.4% in AA and 9.3% ± 3.2% in SNEA, and the effect was significantly higher compared to the HS group—proliferation increased by 4.0% ± 1.6% (*p* < 0.05). Supplementing growth medium with 2% of investigated subjects’ blood serum had a positive effect on ASM cell proliferation. In the AA group, proliferation increased by 23.8% ± 7.0% if ASM cells were cultured alone in serum-supplemented medium and did not significantly different if they were cultured in serum-supplemented medium with eosinophils—proliferation increased by 27.2% ± 8.7%. The same results were received in the SNEA group (proliferation increased by 34.3% ± 10.8% and 28.1% ± 10.4%, *p* < 0.05) and the HS group (increased by 25.4% ± 5.7% and 30.8% ± 4.6%, *p* < 0.05). Moreover, supplementing serum-free growth medium with 25 nM of ascorbic acid significantly decreased ASM proliferation by 36.3% ± 11.6% in AA, 27.4% ± 17.6% in SNEA, and 22.3% ± 12.5% in the HS group. The combined cell culture with eosinophils did not change the ascorbic acid effect on ASM cell proliferation in the AA and SNEA groups (decreased by 33.9% ± 10.7% and 33.6% ± 14.8%, respectively) but removed its negative effect in the HS group (Figure 6A).

We measured the AA and HS eosinophil effect on the proliferation of another structural cell type—Pulmonary fibroblasts. We received similar results—Proliferation significantly increased by 11.4% ± 2.3% in AA and 4.6% ± 1.6% in HS groups after co-culturing with eosinophils. Supplementing growth medium with blood serum also had a positive effect—The pulmonary fibroblast cell number increased by 18.4% ± 5.3% and 10.8% ± 4% in the AA and HS groups, respectively. However, co-culturing with eosinophils in serum-supplemented growth medium increased proliferation by 19.2% ± 4.1% in the AA group, but in the HS group, the effect of eosinophils was the same as in serum-free medium. Different results, compared with co-cultures with ASM cells, were seen by evaluating the effect of ascorbic acid on the proliferation of pulmonary fibroblasts. In the AA group, fibroblasts did not lose their proliferative activity after supplementing growth medium with 25 nM of ascorbic acid; however, a significant decrease (6.5% ± 2.6%) was observed when measuring the effect of HS eosinophils on fibroblast proliferation in ascorbic acid-supplemented growth medium (Figure 6B).

Finally, we investigated how adding different quantities of eosinophils correlate with their effect on structural cell proliferation. We determined that in the AA group, half (1/2x) the number of eosinophils had a similar effect as a typically-used eosinophil number on ASM cell proliferation and increased it by 13.02% ± 4.2%; however, after using twice (2x) the number of eosinophils, the significant pro-proliferative effect was lost. By using pulmonary fibroblasts, the results were different—1/2x and 2x quantity of eosinophils had no positive effect on cell proliferation. In the SNEA group, we used only co-cultures with ASM cells and observed that a 1/2x quantity of eosinophils had no significant proliferative effect, but a 2x quantity increased ASM proliferation by 12.4% ± 5.2% (*p* < 0.05), without significant differences to the effect of a typically-used eosinophil number. In the HS group, we determined that a 1/2x quantity of eosinophils had no effect on ASM cells or pulmonary fibroblast proliferation; however, for a 2x eosinophil quantity, both cases significantly decreased the eosinophil proliferation by 8.5% ± 3.4% and 12.0% ± 5.5%, respectively (*p* < 0.05) (Figure 6C,D).

**Figure 7:**
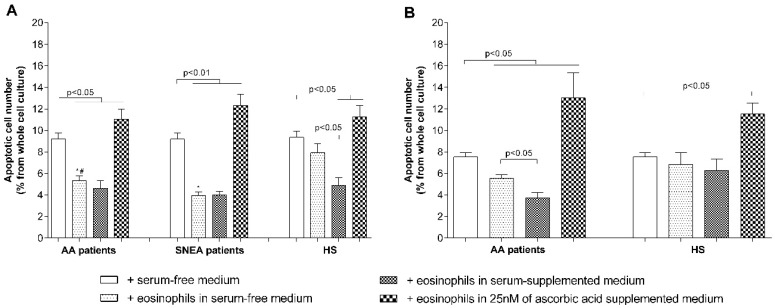
Eosinophils’ effect on pulmonary structural cell apoptosis. (**A**) Apoptosis of ASM cells; (**B**) apoptosis of pulmonary fibroblasts. AA—Allergic asthma, SNEA—Severe non-allergic eosinophilic asthma, HS—Healthy subjects. Results from independent experiments of AA—*n* = 14, HS—*n* = 15, SNEA—*n* = 10. * *p* < 0.05 compared with the healthy eosinophils group; ^#^ *p* < 0.05 compared with the SNEA eosinophils group. Added blood serum—2% of *v*/*v*; ascorbic acid concentration—25 nM. Statistical analysis: between investigated groups—Mann–Whitney two-sided U-test; within one study group—Wilcoxon matched-pairs signed-rank two-sided test.

We investigated the mechanisms through which eosinophils affect pulmonary structural cell proliferation. We measured the eosinophil effect on pulmonary structural cell apoptosis after 24 h of co-culturing. Approximately 9.2% ± 0.6% of ASM cells and 7.5% ± 0.4% of pulmonary fibroblasts in culture are apoptotic after culturing and detachment procedures. After co-culturing with eosinophils, the apoptotic ASM cell number significantly (*p* < 0.05) decreased to 5.3% ± 0.5% in the AA patients group, to 4.0 ± 0.3 in the SNEA patients group (*p* < 0.05 compared with AA), but had no significant effect (*p* = 0.14) in the HS group. Co-culturing with eosinophils reduced the number of apoptotic pulmonary fibroblasts in the AA patient group by 5.5% ± 0.4% (*p* < 0.05) but had no effect in the HS group. Supplementing growth medium with blood serum (2% of *v*/*v*) of the investigated individuals enhanced the effect of eosinophils, and the apoptotic ASM cell number decreased from 7.9% ± 0.9% in serum-free medium to 4.9% ± 0.7% in serum-supplemented medium (*p* < 0.05). Unlike in the ASM cells group, supplementing growth medium with blood serum enhanced the effect of eosinophils on pulmonary fibroblasts only in the AA patients group—the apoptotic number reduced from 5.5% ± 0.4% to 3.7% ± 0.5% of the total cell count in culture. Moreover, considering that ascorbic acid is a well-known antioxidant and can eliminate the effect of the released reactive oxygen species of eosinophils on ASM cells or pulmonary fibroblast apoptosis, we supplemented the growth medium with a minimum of 25 nM of final concentration of ascorbic acid and obtained the result that the apoptotic ASM cell number increased to 11.1% ± 0.9% in the AA group, to 12.3% ± 1.1% in the SNEA patient group, and 11.2% ± 1.1% in the HS group (*p* < 0.05), as well as the pulmonary fibroblast number to 13.0% ± 2.4% in the AA patient group and 11.5% ± 1.0% in the HS group (*p* < 0.05) (Figure 7).

### 3.4. The Effect of Bronchial Allergen Challenge to Eosinophil Activity

The bronchial challenge with *D. pteronysinnus* allergen was performed for 11 individuals from the AA patient group and 11 individuals from the HS group. The effect of in vivo allergen provoked disease exacerbation to eosinophil activity and was evaluated by comparing the results before and 24 h after a bronchial allergen challenge of the same study subject. A significant increase was observed in the peripheral blood eosinophil count in the AA group following allergen exposure from 0.38 ± 0.08 × 10^9^/L to 0.45 ± 0.06 × 10^9^/L of cells, without significant changes in the HS group.

**Figure 8:**
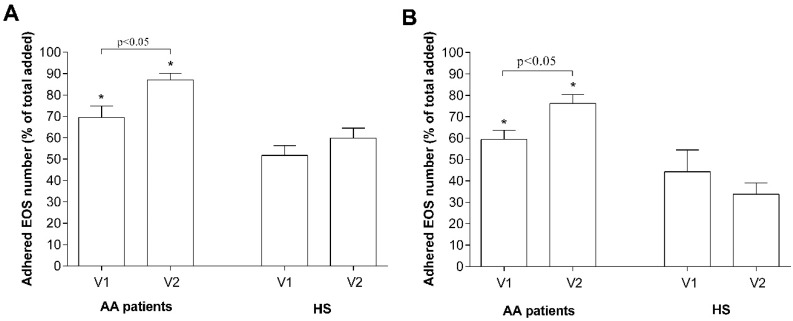
Bronchial allergen challenge effect on the efficiency of eosinophil adhesion. (**A**) Eosinophils adhesion in co-culture with ASM cells; (**B**) eosinophils adhesion in co-culture with pulmonary fibroblasts. Results from independent experiments of AA—*n* = 11, HS—*n* = 11; EOS—Eosinophils, AA—Allergic asthma, HS—Healthy subjects. * *p* < 0.05 compared with the adhesion of the healthy eosinophil group. V1—Visit 1 (before bronchial allergen challenge); V2—Visit 2 (24 h after bronchial allergen challenge). Statistical analysis: between investigated groups—Mann–Whitney two-sided U-test; within one study group—Wilcoxon matched-pairs signed-rank two-sided test.

Eosinophil adhesion 24 h after a bronchial allergen challenge increased only in the AA group with no effect on HS eosinophils. The number of adhered eosinophils in co-cultures with ASM cells increased from 69.5% ± 5.4% to 87.06% ± 3.1% and in co-cultures with pulmonary fibroblasts from 59.4% ± 4.3% to 76.2% ± 4.2% of the total added eosinophil count (*p* < 0.05) (Figure 8).

**Figure 9:**
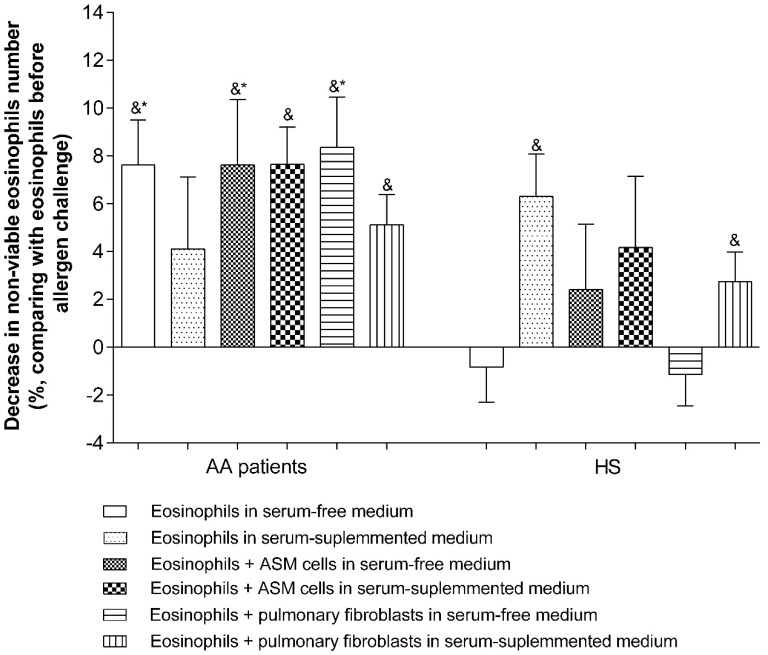
The bronchial allergen challenge effect on the number of non-viable eosinophils. Results from independent experiments of AA—*n* = 11, HS—*n* = 11; AA—Allergic asthma, HS—Healthy subjects. * *p* < 0.05 compared with the viability of the healthy eosinophil group; ^&^ *p* < 0.05 compared with the viability of eosinophils before the bronchial challenge. Added blood serum—2% of *v*/*v*. Statistical analysis: between investigated groups—Mann–Whitney two-sided U-test; within one study group—Wilcoxon matched-pairs signed-rank two-sided test.

A bronchial allergen challenge activated eosinophils in vivo and had a positive effect on AA patients eosinophils viability—The number of non-viable eosinophils decreased by 7.6% ± 1.8% if eosinophils were incubated alone or in serum-free growth medium (*p* < 0.005), but had no effect on healthy eosinophils. However, by using in vivo allergen-activated eosinophils and serum-supplemented growth medium, a positive effect was only obtained in the HS group (non-viable eosinophils number decreased by 6.3% ± 1.8%, *p* < 0.01), but had no effect on the AA patient group compared with non-activated eosinophils. Moreover, AA patients’ allergen-activated eosinophil viability was increased if they were co-cultured with pulmonary structural cells in serum-free medium (non-viable eosinophils’ number decreased by 7.6% ± 2.7% in co-culture with ASM cells, and by 8.3% ± 2.1% in co-culture with pulmonary fibroblasts, *p* < 0.01). Co-culturing in investigated subjects’ blood serum-supplemented growth medium did not have any further effect on eosinophil viability (Figure 9).

**Figure 10:**
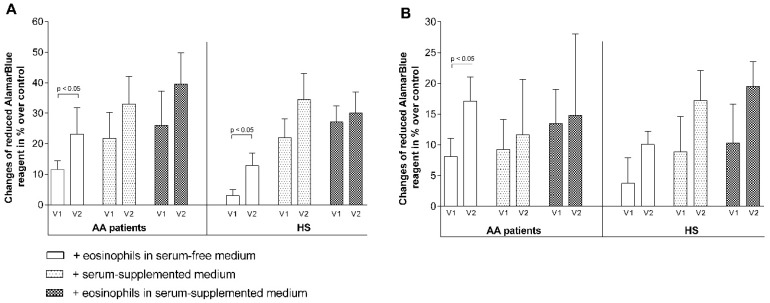
Bronchial allergen challenge effect on pulmonary structural cell proliferation. (**A**) The effect of a bronchial allergen challenge on eosinophil-promoted ASM cell proliferation; (**B**) the effect of a bronchial allergen challenge on eosinophil-promoted pulmonary fibroblast proliferation. Results from independent experiments of AA—*n* = 11, HS—*n* = 11; V1—Visit 1 (before the bronchial allergen challenge); V2—Visit 2 (24 h after the bronchial allergen challenge). Statistical analysis: between investigated groups—Mann–Whitney two-sided U-test; within one study group—Wilcoxon matched-pairs signed-rank two-sided test.

Moreover, we evaluated in vivo the effect of activated AA and HS eosinophils on structural cell proliferation. We observed that eosinophils’ pro-proliferative effect on ASM cells after a bronchial allergen challenge was enhanced by 11.6% ± 8.7% in the AA group and by 9.8% ± 4.1% in the HS group, compared with the effect of non-activated eosinophils. An enhanced pro-proliferative effect on pulmonary fibroblasts was only observed in the AA group (enhanced by 7.2% ± 2.5%), compared with the effect of non-activated eosinophils (Figure 10).

**Figure 11:**
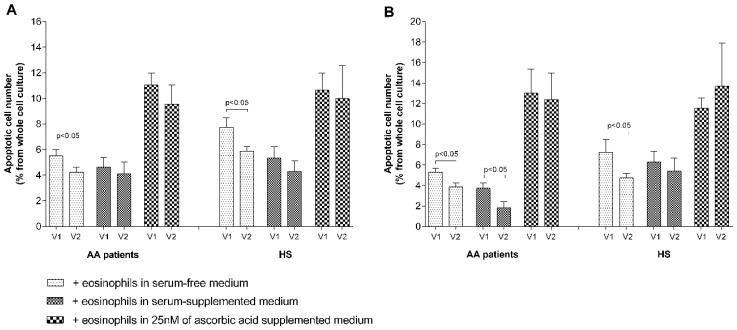
The effect of a bronchial allergen challenge on pulmonary structural cell apoptosis. (**A**) The effect of a bronchial allergen challenge on eosinophil-induced ASM cell apoptosis. (**B**) The effect of a bronchial allergen challenge on eosinophil-induced pulmonary fibroblast apoptosis. Results from independent experiments of AA—*n* = 11, HS—*n* = 11; V1—Visit 1 (before the bronchial allergen challenge); V2—Visit 2 (24 h after the bronchial allergen challenge). Added blood serum—2% of *v*/*v*; ascorbic acid concentration—25 nM. Statistical analysis: between investigated groups—Mann–Whitney two-sided U-test; within one study group—Wilcoxon matched-pairs signed-rank two-sided test.

Finally, a bronchial allergen challenge enhanced the effect of eosinophils on the reduction of ASM cells and pulmonary fibroblast apoptosis. The apoptotic ASM cell number reduced from 5.5% ± 0.5% of the total cell count in culture, to 4.2% ± 0.4% in the AA patient group, and from 7.7% ± 0.7% to 5.8% ± 0.3% in the HS group. Meanwhile, the number of apoptotic pulmonary fibroblasts reduced from 5.3% ± 0.4% to 3.9% ± 0.4% in the AA patient group, and from 7.2% ± 1.3% to 4.7% ± 0.5% in the HS group. After supplementing growth medium with the blood serum of the investigated individuals—collected after the bronchial allergen challenge—the effect of eosinophils on the reduction of apoptosis in pulmonary fibroblasts was only enhanced in the AA patients group—the apoptotic cell number decreased from 3.7% ± 0.5% in serum-free medium to 1.8% ± 0.6% in serum-supplemented medium (*p* < 0.05) (Figure 11).

## 4. Discussion

Increased eosinophil adhesion and prolonged viability in asthma could be the reason for the increased eosinophil number in asthmatic lungs because of their delayed migration to the bronchial lumen, which contributes to more intense development of airway remodeling. In this study, we found that adhesion of eosinophils of AA and SNEA patients to ASM cells or pulmonary fibroblasts was increased when compared to the HS group. Adhesion to pulmonary structural cells had a significant effect on prolonging the viability of eosinophils in all the investigated groups; however, the highest effect was observed in SNEA patients. Moreover, serum-activated eosinophils from AA and SNEA patients demonstrated an enhanced pro-proliferative effect on pulmonary structural cells. Furthermore, eosinophils from SNEA patients had a more pronounced effect on reducing apoptosis of ASM cells and pulmonary fibroblasts, however, with a similar pro-proliferative effect when compared with eosinophils from patients with AA. In the AA group, in vivo allergen-activated eosinophils demonstrated higher adhesion, viability, and pro-proliferative effects on pulmonary structural cells compared to non-activated eosinophils.

Airway eosinophilia is associated with more frequent exacerbations of asthma, which contributes to the development of airway remodeling [34,35,36]. Eosinophil infiltration from the circulation into the asthmatic airway depends on the activation sites of eosinophils, which leads to their arrest on activated endothelium, extravasation into the airway wall, and migration through airway tissues into the airway lumen. Airway eosinophils demonstrate increased activity of two main eosinophils integrins—α_4_β_1_ and α_M_β_2_ [37]; moreover, blood eosinophil integrins are found to be in a more-activated state during asthma [38]. Previously, we demonstrated that increased expression of eosinophil integrins in asthma leads to increased eosinophil adhesion and is associated with eosinophil-induced airway remodeling [22,23]. In the current study, we showed that in the bloodstream of the HS, between 40% and 50% of eosinophils exist, which could rapidly (within the hour) adhere onto other cell or ECM proteins, while during asthmatic conditions, this number increases to 60–70%, with no significant difference between AA and SNEA phenotypes (Figure 4A,B). Moreover, pre-activation of isolated eosinophils with mediators found in blood serum does not affect their adhesion properties; on the contrary, blood serum from SNEA patients reduced the maximum number of adhered eosinophils, probably because of the use of inhaled steroids. It demonstrates that eosinophils do not lose their activity during the isolation processes. Furthermore, eosinophil degranulation, assessed by EPO activity, did not statistically differ between the investigated groups; however, the EPO activity of eosinophils of SNEA patients is prone to decrease (Figure 4C).

Eosinophils adhere when their integrins recognize and connect to counter-receptors on other cells or ligands in the ECM proteins. However, there is a limited number of these counter-receptors that could restrict eosinophil adhesion. We showed that after increasing the number of more-adhesive eosinophils from AA and SNEA patients in the co-cultures, the ratio of adhered eosinophils to total added eosinophils significantly decreased and became the same as in the HS group (Figure 4A,B). We assumed that the management of pulmonary structural cell adhesion molecules and ECM component expression could play an important role by regulating eosinophil-induced airway structural changes. Increased eosinophil adhesion intensity could be explained by increased expression of outer membrane integrins [22] or by their different activation states [37,39]. Integrins exist in an inactive bent, an intermediate-activity extended closed, and a high-activity extended open conformation, and in that way modulate eosinophil adhesion and migration [40,41]. The severity of the disease or disease exacerbation could affect eosinophil’s integrin expression and its activation states, contributing to eosinophil’s further pro-inflammatory effect. However, it requires expanded investigation in a background of different asthma phenotypes and allergen-induced eosinophil activation.

Eosinophil’s contribution to airway remodeling in asthma depends not only on its increased infiltration, but on its survivability in airways as well, which prolongs the effect of eosinophils on pulmonary structural cells. It was primarily described from several scientist teams, who revealed the importance of direct contact with pulmonary structural cells to their survivability, probably via signaling through GM-CSF and IL-1β [12,13,14,15]. Circulating eosinophils are contained in the mixture of various mediators found in peripheral blood, which regulates eosinophils’ activation and survival. We found that AA and SNEA patients‘ eosinophils are characterized by greater survival compared with those of HS; moreover, the highest eosinophil viability was observed in the SNEA group (Figure 5). As eosinophils were incubated in serum-free growth medium, this demonstrated that SNEA patients‘ eosinophils had the strongest cytokine-induced survivability signals in peripheral blood. If eosinophils were incubated in investigated individual serum-supplemented medium, AA eosinophils’ survival significantly increased and remained higher when compared with HS eosinophils. Blood serum enhanced eosinophils viability, thus demonstrating the healing process of isolated eosinophils if they are not pre-activated by mediators found in asthmatic blood serum. However, SNEA patients‘ blood serum had no effect on eosinophils’ survival, probably due to fully-occupied survivability regulating receptors after eosinophil activation in vivo, or medications present in the blood serum (Figure 5).

Activation of eosinophilopoietin receptors [42] may not be the only factors regulating eosinophil viability. Adhesion through integrins can also be understood as a survival signal [43]. Incubation for 24 h with ASM cells or pulmonary fibroblasts significantly increased AA and SNEA patients’ eosinophil viability, compared with eosinophils cultured alone, and the highest effect was observed in the SNEA patient group. This highlights the importance of contact with the opposite cell or their released extracellular matrix proteins on eosinophils’ viability. Moreover, eosinophils equally adhere to ASM cells and pulmonary fibroblasts without preference for one cell type (Figure 4). However, more detailed research is needed to understand whether eosinophils adhere more to adhesion molecules on the surface of structural cells, or to specific sites on ECM proteins, and how this determines eosinophils’ viability. Furthermore, a longer incubation period between eosinophils and structural cells should be used to find out how long eosinophils can stay viable for with or without an external stimulus in different asthma phenotypes.

Asthma-related airway remodeling mostly involves the airway epithelium, ASM, and extracellular matrix components [44]. As the number of eosinophils in asthmatic airways is enhanced, their role in disturbing local homeostasis is indisputable. It is known that ASM cells proliferate more during asthma; however, there is only minimal research showing that eosinophils influence this process [21,22,23]. Likewise, there is lack of information regarding the effect of eosinophils on pulmonary fibroblast proliferation [24], which could significantly contribute to ECM remodeling in asthma, as well as how eosinophils of SNEA patients affect pulmonary structural cell proliferation. When eosinophils migrate to the airways, the surrounding mediators might change, and further activation of eosinophils mostly depends on their activation in peripheral blood or pre-activation by released mediators of pulmonary structural cells. In the current study, we investigated the effect of eosinophils on ASM cells or pulmonary fibroblast proliferation in the context of individuals’ blood serum that might maintain the initial activation of eosinophils. Our results demonstrated that eosinophils, isolated from SNEA patients, have the same effect on ASM cell proliferation as eosinophils isolated from AA patients. Moreover, both ASM cells and pulmonary fibroblasts respond similarly to the pro-proliferative effect of eosinophils (Figure 6A,B).

There are many mechanisms through which cell proliferation can be promoted [45]. One of the mechanisms revealed in this study demonstrated that eosinophils significantly inhibit pulmonary structural cell apoptosis in AA and SNEA groups, but not in HS (Figure 7A,B); however, the precise mechanism is unknown. Blood serum is important for structural cell proliferation and had a higher pro-proliferative effect compared with eosinophils in all the investigated groups. However, there is no exact information regarding the concentrations of mediators in the surrounding ASM cells and the pulmonary fibroblast environment in vivo, which could be different from those used in vitro. Apoptosis measurements also demonstrated that blood serum does not enhance the effect of eosinophils on reducing ASM cell apoptosis in the AA and SNEA groups, as these eosinophils did not lose their primary activity in peripheral blood after 24 h of incubation. However, less-activated eosinophils in the HS group were pre-activated by blood serum and demonstrated a more pronounced effect in reducing structural cell apoptosis (Figure 7A). 

Eosinophils release not only remodeling-related mediators [1] but could also be toxic to many tissues because of released cytotoxic cationic proteins [46]. The best known and most accessible antioxidant is ascorbic acid, in which the blood levels are linked to asthma pathogenesis and its prevention [47,48]. A concentration of more than 100 nM ascorbic acid could be toxic to many cells while demonstrating a lower proliferative effect [49]. We used a concentration of only 25 nM ascorbic acid, avoiding possible changes in growth-medium pH levels. However, our data showed that ascorbic acid alone significantly reduced ASM cells, but not pulmonary fibroblast proliferation, which could be partially explained by the close interface between ascorbic acid and collagen synthesis [50]. In combination with eosinophils, a negative ascorbic acid effect on ASM cells was eliminated only in the HS group, but was evidenced for pulmonary fibroblasts. Moreover, eosinophils in ascorbic acid-supplemented medium significantly increased ASM cells and pulmonary fibroblast apoptosis in all the investigated groups (Figure 6A,B). However, there is no clear explanation of these results according to the literature data, and therefore more research should be done with different ascorbic acid concentrations and cell densities in co-cultures.

Eosinophils have two sides to their biological role that could be partly explained by existing distinct eosinophil phenotypes in peripheral blood and lung tissues [51] with different biological roles. One phenotype is inflammation-related, another one has a greater effect on the remodeling processes. It shows that in the AA, SNEA, and HS groups there can exist different proportions of eosinophil phenotypes; therefore, an increased or decreased number of eosinophils in co-cultures disbalance the effect of the predominant phenotype effect (Figure 5C,D). In the AA group, a twofold reduced number of eosinophils had the same proliferative effect on ASM cells, but the effect was lost to pulmonary fibroblasts; however, a twofold increased number of eliminated eosinophils induced ASM cells and pulmonary fibroblast proliferation, probably due to an increased effect of the inflammation-related eosinophil phenotype. In the SNEA group, a reduced number of eosinophils had no effect on ASM proliferation, while normal and increased numbers of eosinophils had a similar positive effect. In the HS group, the reduced eosinophil number eliminated their effect on ASM cells and pulmonary fibroblast proliferation; however, a twofold increase in the number of eosinophils significantly reduced structural cell proliferation. Different ratios of eosinophil phenotypes in peripheral blood during AA, SNEA, and HS could explain these findings. In the HS group, as there should be less of specific disease-related signals for the attraction of homeostatic eosinophils, the predominant phenotype might be more inflammation-related eosinophils, which started to dominate after an increase in their count. With the SNEA group, the predominant phenotype was remodeling-related homeostatic eosinophils, and their proliferative effect correlated with their count, whereas in AA, the ratio should have been intermediate and increased the number of added eosinophils, eliminating the proliferation-promoting effect. However, the number of inflammatory eosinophils was not enough to reduce this effect (Figure 5C,D). Moreover, there is a lack of studies about different eosinophil phenotypes; therefore, more data is needed to confirm these results.

Asthma is a heterogeneous disease with multiple possible targets in its pathogenesis. AA severity is associated with the frequency of exacerbations after exposure to allergens that may contribute to the development of airway remodeling [52]. We sought to find out the role of eosinophils during an acute asthma episode, which is determined only by their quantitative differences or can be characterized by significant changes in their activity. During an allergen attack, released alarmins promote Th2 cells to produce eosinophilopoetins that may affect the number and functions of eosinophils [53]. Allergen-induced late asthmatic responses are mainly described by an increased number of airway inflammatory cells; however, the exact changes in eosinophil activity under allergen-induced disease exacerbation are mostly unknown. Our team previously demonstrated that during allergen-induced late-phase airway inflammation, peripheral blood eosinophils demonstrated further alterations of their functional activity, manifested by enhanced spontaneous reactive oxygen species production, increased chemotaxis, and diminished apoptosis in patients with AA [54]. Our findings show that the exposure of allergens activates AA patients’ eosinophils in vivo, or they are released from bone marrow in a more activated state. They demonstrated increased adhesion and survivability properties and confirmed that after an acute episode, released eosinophils can survive in an asthmatic airway for a longer period (Figure 7 and Figure 8). Moreover, allergen exposure had an effect on HS eosinophils, also promoting their viability, although subjects from the HS group were not sensitized to *D. pteronyssinus.* We used one of the most common home dust mite allergens with which the whole human population frequently comes into contact. It allowed us to assume that after a constant natural exposure of *D. pteronyssinus,* a memory of this allergen develops. The reaction of organisms to the high doses of inhaled concentrated allergen is too weak for bronchoconstriction; however, it is enough to slightly stimulate type-2 inflammation and activate eosinophils. This is important for future investigations, which should aim to understand possible AA development later in life.

More-enhanced eosinophil activity during allergen-induced asthma exacerbation required more intense disease treatment. Allergen-activated eosinophils demonstrated a twofold increased effect to ASM cells and pulmonary fibroblast proliferation via reduced apoptosis (Figure 9 and Figure 10). It demonstrates that asthma exacerbation is associated with more intense development of airway remodeling via eosinophils’ pro-proliferative effect. Tang and colleagues discovered that recruitment of eosinophils into asthmatic lungs during allergen-induced airway responses proceed via the IL-25/IL-25R axis and IL-25 neutralization and may be a potential therapeutic target for the attenuation of allergen-induced asthmatic responses mediated by airway eosinophilia [55]. However, there exists other well-known alarmins, such as IL-33 or thymic stromal lymphopoietin, that could also contribute to the recruitment of eosinophils into asthmatic lungs. Our findings suggest that eosinophil adhesion is important for their activity and effect on pulmonary structural cells; therefore, inhibiting their adhesion properties, together with chemotaxis, could be an effective way of attenuating their negative role during different asthma phenotypes and disease exacerbations.

## 5. Conclusions

Increased adhesion of eosinophils prolonged their viability, and might be related to enhancing their pro-proliferative effect on ASM cells and pulmonary fibroblasts in asthma. Moreover, eosinophils from SNEA patients demonstrated higher viability and inhibition of pulmonary structural cell apoptosis compared to the AA group, while the adhesive properties and pro-proliferative effects were similar for both. In the AA group, in vivo allergen-activated eosinophils presented enhanced adhesive properties, viability, and a pro-proliferative effect on pulmonary structural cells compared to non-activated eosinophils. These results could be important in the development of new therapeutic tools for the suppression of eosinophil functions in asthma, focusing not only on eosinophils’ depletion but also on their survivability.

## Figures and Tables

**Table 1:** Demographic and clinical characteristics of the study population.

	Healthy Subjects	SNEA Patients	AA Patients
Number, *n*	15	10	14
Sex, M/F	3/12	4/6	7/7
Age, years	28.4 ± 1.8	46.5 ± 3.0 ^#,^*	28.0 ± 2.3
BMI, kg/m^2^	23.2 ± 1.2	26.9 ± 2.2	24.7 ± 1.6
PD_20M_, geometric mean (range), mg	NR	ND	0.08 (0.02–0.26)
PD_20A_, geometric mean (range), HEP/mL	NR	ND	0.84 (0.15–3.04)
IgE, IU/mL	29.5 ± 6.0	147.2 ± 30.7 *	202.8 ± 41.9 *
FEV_1_, L	3.9 ± 0.18	2.0 ± 0.4 ^#,^*	3.9 ± 0.2
FEV_1_, % of predicted	106.6 ± 3.7	57.6 ± 8.6 ^#,^*	95.9 ± 3.3
Blood eosinophil count, ×10^9^/L	0.18 ± 0.02	0.52 ± 0.11 ^#,^*	0.36 ± 0.07 *
FeNO, ppb	12.9 ± 1.5	44.9 ± 10.7 *	59.5 ± 11.5 *

AA—Allergic asthma; SNEA—Severe non-allergic eosinophilic asthma; F—Female; M—Male; FEV_1_—Forced expiratory volume in 1s; PD_20M_—The provocation dose of methacholine causing a 20% decrease in FEV_1_; PD20A—the provocation dose of allergen D. pteronyssinus causing a 20% decrease in FEV1; BMI—Body mass index; FeNO—Fractional exhaled nitric oxide; IgE—Immunoglobulin E; NR—Not responded; ND—Not done. Data presented as the mean ± standard error of the mean, except PD_20M_ and PD_20A_ which are provided as geometric mean (range). * *p* < 0.05 compared with HS group; ^#^ *p* < 0.05 compared with AA group. Statistical analysis—Mann–Whitney two-sided U-test.
